# Conducting Research in Disease Outbreaks

**DOI:** 10.1371/journal.pntd.0000335

**Published:** 2009-04-28

**Authors:** Ruth Macklin, Ethan Cowan

**Affiliations:** 1 Department of Epidemiology and Population Health, Albert Einstein College of Medicine, Bronx, New York, United States of America; 2 Department of Emergency Medicine, Albert Einstein College of Medicine, Bronx, New York, United States of America; New York Blood Center, United States of America

Conducting research in an emergency situation, such as an outbreak of disease, poses ethical challenges. These challenges differ according to the type of research: epidemiologic or clinical, and for the latter, whether the disease outbreak can be anticipated in advance. We address these three situations, proposing different potential solutions for each.

In an outbreak situation, public health authorities undertake a rapid response in an effort to document the existence and magnitude of a public health problem in the community and to implement appropriate measures to address the problem [1]. This rapid response will in some cases preclude the possibility of clearance by a research ethics committee since the time required to develop and submit a detailed research protocol and respond to any requested modifications by the committee, followed by re-review, would thwart the very purpose of the response. According to one prominent view, to require a full written protocol and submission to an ethics review board would not be in the interests of the individuals or the community because the resulting delays would frequently cause excess disease and death [2]. These authors suggest, however, that emergency response consent forms could be developed and used in these situations. Other individuals engaged in public health practice have voiced concern that subjecting their work to the routinely required “regulatory constraints imposed on research” would prevent flexible and timely approaches to situations such as disease outbreaks [3]. They argue that timeliness is a major requirement that would have to be counterbalanced with other ethical concerns. An example is that of pandemic influenza outbreaks, in which it is alleged that the review process would impair the ability of public health agencies to react in a timely manner.

For researchers and public health agencies, therefore, the question is how to comply with the ethical requirement that research be approved by a properly constituted, independent ethical review committee (ERC) but still enable a prompt response when an outbreak occurs. If we concede that existing methods of ethical review are too protracted to be useful in outbreak situations, can alternative mechanisms be employed to ensure that such investigations undergo some type of ethical review? How can the rights and welfare of individuals be protected during investigations of disease outbreaks, and at the same time enable such investigations to be carried out expeditiously?

In addition to review by a research ethics committee, a fundamental ethical requirement in research is to obtain informed consent from participants. Although not all research requires the informed consent of individual subjects, the vast majority of clinical research, most social and behavioral research, and some epidemiologic research must be carried out with the voluntary, informed consent of participants or their legally authorized representatives. In contrast, in many instances of public health practice, collection and use of information or human biological specimens can be conducted without a written informed consent document and without obtaining permission to store the samples for future use. However, a problem could arise if an investigator wants to use these samples, collected initially for public health purposes, for research. Current research practice calls for obtaining consent for the use of specimens in future research that may not be known at the time they are collected. Samples obtained in a non-research context without such provisions may require going back to get consent from sampled individuals, which could be logistically difficult, if not impossible. For example, samples with no identifying information could not be traced back to the individuals from whom they were obtained. Additionally, if a considerable time has elapsed between the collection of identifiable samples and the plan to use them in research, it could be difficult to locate the individuals. If researchers intend to use previously collected samples without going back to get consent, they must provide a justification in the protocol submitted to the research ethics committee.

It is hard to see, even in non-research contexts, how human biological specimens could be obtained or even how individuals could be surveyed without first obtaining their permission to draw blood or to ask questions that may intrude on their privacy. Nevertheless, procedures for obtaining consent from individuals in an outbreak situation could depart from those typically used in other investigations and still be ethically acceptable. It is surely not necessary to include all 26 items listed as “essential information for prospective research subjects” in the CIOMS *International Ethical Guidelines for Biomedical Research* [4]. It is important, however, for investigators to ensure that individuals understand that what they are consenting to is research, and not routine activities carried out by public health practitioners during a disease outbreak. Nevertheless, ensuring a participant's understanding of the difference between public health practice and research may be complicated by the emotional distress that potential participants can experience during an outbreak situation. Although appropriate steps are needed when participants become vulnerable in such situations, that would not change the level of risk from minimal to more than minimal risk. The level of risk is determined by the procedures in the research proposal and not by the characteristics of the population. When populations are vulnerable for whatever reason, the approach to participants in research requires establishing safeguards or added protections. Such safeguards can range from specifying exclusion criteria for potential subjects in great distress to taking steps to work sensitively to protect the rights and interests of marginalized populations. An institutional review board (IRB) chair or designated reviewer can always request additional reviewers to ensure that vulnerable individuals are adequately protected.

## The US Food and Drug Administration “Emergency” Rules

It might be thought that the problems related to obtaining consent from people in an outbreak situation could be overcome by an appeal to the clause in the US Code of Federal Regulations that permits a waiver of informed consent for research conducted in an emergency, the so-called final rules [5]. However, the rule is inapplicable to the situation under discussion here. The rule is intended to apply to clinical situations, such as major trauma, cardiac arrest, or other incapacitating circumstances, in which prospective research participants are unable to grant consent. Under the rules, the situation must be life-threatening, requiring an immediate intervention, and there must be no standard treatment that could be used instead of an experimental intervention. Consent may be waived only when next of kin or a guardian is not present or cannot be reached in sufficient time before initiating the research. It is clear then, that the exception from informed consent requirements applies only to research in which individual patients in life-threatening situations must receive immediate treatment, and not the type of research conducted in an emergency response to an outbreak of infectious disease.

In addition, nothing in the Food and Drug Administration (FDA) final rules exempts the research from review by an IRB or ERC. On the contrary, the IRB review and logistical requirements needed for approval of studies under the final rules have been described as so burdensome that they may be impeding much needed resuscitation research [6],[7]. One reason studies that fall under the umbrella of the final rules are not exempt from IRB review is that they involve a foreseen and common emergency situation where there is ample time to prepare a full research protocol and have it approved by a committee. Potential participants in these studies are unfortunate but not unexpected members of communities where the disease or occurrence in question (e.g., major trauma, cardiac arrest) takes place at some known frequency.

Another requirement in the FDA rules is community consultation before the research can be initiated. A plan for such consultation must be included in the protocol submitted to the IRB. Clearly, in the case of most outbreaks, affected communities probably cannot be identified in advance, precluding the very possibility of conducting such research. For all these reasons, the FDA emergency rules are inapplicable to research conducted in disease outbreaks.

## Possible Solutions

What solutions are available to ensure that public health research in disease outbreaks can proceed without undue delays and yet protect the rights and welfare of human beings who are surveyed, whose blood is drawn, or who receive experimental or off-label drugs? It might be argued that approval of a proposed investigation by a Ministry of Health can serve this purpose. However, approval by a Ministry of Health is not the equivalent of ethical review and clearance by a duly constituted committee. Both types of approval are necessary in research in non-emergency situations, but a governmental office is not equipped to do the same sort of review as a committee with expertise and experience in research ethics.

An appropriate solution would be to seek an alternative mechanism to that of full review of a complete research protocol by an IRB or ERC. Different strategies would be appropriate for epidemiologic research, on the one hand, and clinical trials, on the other.

### Epidemiologic Research

The methodology used in epidemiology can be the same whether an activity is characterized as research or public health practice. In regard to research, a well-established procedure for ethics review already exists that would apply to almost all epidemiologic research conducted in a disease outbreak. That mechanism is expedited review by an ERC. Such investigations pose no more than minimal risk, as the most invasive procedures involving human beings are likely to be blood drawing and survey completion. Expedited review is typically conducted by committee chairs or someone they designate, and can be accomplished within a day or two. Committees could establish a policy for disease outbreak investigations in which a full, detailed protocol need not be submitted. A shorter document describing the background, the purpose of the research, informed consent procedures, and steps to protect the confidentiality of information obtained from the individuals should be acceptable in such a policy. As for the need to obtain informed consent from participants, in epidemiologic research, the requirements for informed consent typically are guided by whether identifiable information is collected and how it is collected. A duly constituted oversight body could decide to waive the requirement for signed consent forms in favor of oral consent depending on the specifics of a research proposal.

### Clinical Research

Research involving experimental medications or new uses for approved drugs is considered more than minimal risk, and therefore cannot be reviewed by the expedited mechanism. Two situations call for somewhat different solutions. The first situation is that of repeat occurrences of an outbreak of a known disease, such as cholera [8],[9],[10]. Although the exact time when an outbreak will occur may not be known, it is well established in certain areas that future outbreaks are highly likely. An example is Tanzania, where cholera outbreaks have occurred regularly since the epidemic was first identified in 1978 [11]. In this situation, investigators can prepare what we shall call a “model protocol,” with all the basic elements spelled out in detail. The model protocol can be submitted for full review to the IRB or ERC, omitting items that are specific to the time and place of the predicted outbreak. When the outbreak occurs, investigators can complete the specific information for review by the committee. Although those specific details could be reviewed in an expedited manner, a problem could arise where the drugs to be studied are not known in advance. Some drugs have higher risk profiles than others, so studies involving such drugs would probably require full committee review.

The second situation, somewhat more problematic, is an outbreak of a disease heretofore unknown. Probably the best recent example is that of severe acute respiratory syndrome (SARS) [12]. The novelty of SARS, and the fact that the epidemic was both rapid in its onset and short in its duration, made it difficult to study. Despite the global impact of SARS, relatively few treatment protocols were studied in clinical trials [13],[14],[15],[16]. One study of early corticosteroid treatment for SARS was even terminated prematurely because the epidemic had subsided just as the study got underway [14].

The SARS epidemic highlights the major difficulties in conducting clinical research in disease outbreaks involving novel disease-causing agents. Unlike a disease like cholera, where an outbreak and a population can be predicted with some certainty, SARS occurred in a population that was impossible to pre-identify. Furthermore, the causative agent of SARS was initially unknown, and no existing approved treatment protocols were in place. Despite the logistical and methodological difficulty of conducting clinical trials in outbreaks of new diseases, it is precisely these situations where clinical studies are most needed. Unfortunately, the use of “model protocols” to enable the more rapid IRB or ERC review described above may not be sufficient for outbreaks of new diseases. However, in the case of pandemic influenza, a model protocol could still be developed even though the particular strain of the virus may not be known in advance. Existing antiviral medications could be used in initial studies until new preventive vaccines or therapeutic medications can be manufactured and used in subsequent clinical trials.

The most urgent concern at the time of an outbreak of any disease is to implement public health measures to contain its spread. What those measures should be, and to what extent they may involve limitations on individual liberty and other social distancing mechanisms, pose different ethical challenges. We have described a mechanism whereby epidemiologic research can commence immediately, under the conditions of expedited review outlined above. In the meantime, investigators will have to develop clinical research protocols to address a previously unknown disease and seek IRB approval in the usual manner or through the use of “model protocols.”

## Conclusion

Some form of ethical oversight is needed to conduct an investigation of a disease outbreak, be it predictable or unanticipated. The mechanism and procedures can vary from that of an established ERC, acting in an expedited manner for minimal risk research, to development of a model protocol submitted to a committee for full review in advance of an anticipated future outbreak. Such safeguards can help to ensure that the rights and welfare of individuals are protected in disease outbreaks and that communities maintain trust in public health research and practice.
